# Clinical and mechanism advances of neuronal intranuclear inclusion disease

**DOI:** 10.3389/fnagi.2022.934725

**Published:** 2022-09-13

**Authors:** Yueqi Liu, Hao Li, Xuan Liu, Bin Wang, Hao Yang, Bo Wan, Miao Sun, Xingshun Xu

**Affiliations:** ^1^Department of Neurology, The First Affiliated Hospital of Soochow University, Suzhou, China; ^2^Institute of Neuroscience, Soochow University, Suzhou, China; ^3^Institute for Fetology, The First Affiliated Hospital of Soochow University, Suzhou, China; ^4^Jiangsu Key Laboratory of Neuropsychiatric Diseases, Soochow University, Suzhou, Jiangsu, China

**Keywords:** neuronal intranuclear inclusion disease, neurodegenerative diseases, inflammation, NOTCH2NLC, nucleotide repeat expansion disorders

## Abstract

Due to the high clinical heterogeneity of neuronal intranuclear inclusion disease (NIID), it is easy to misdiagnose this condition and is considered to be a rare progressive neurodegenerative disease. More evidence demonstrates that NIID involves not only the central nervous system but also multiple systems of the body and shows a variety of symptoms, which makes a clinical diagnosis of NIID more difficult. This review summarizes the clinical symptoms in different systems and demonstrates that NIID is a multiple-system intranuclear inclusion disease. In addition, the core triad symptoms in the central nervous system, such as dementia, parkinsonism, and psychiatric symptoms, are proposed as an important clue for the clinical diagnosis of NIID. Recent studies have demonstrated that expanded GGC repeats in the 5′-untranslated region of the NOTCH2NLC gene are the cause of NIID. The genetic advances and possible underlying mechanisms of NIID (expanded GGC repeat-induced DNA damage, RNA toxicity, and polyglycine-NOTCH2NLC protein toxicity) are briefly summarized in this review. Interestingly, inflammatory cell infiltration and inflammation were observed in the affected tissues of patients with NIID. As a downstream pathological process of NIID, inflammation could be a therapeutic target for NIID.

## Introduction

Neuronal intranuclear inclusion disease (NIID) was first described in 1968 and is considered to be a rare neurodegenerative disease (Lindenberg et al., 1968). With the development of imaging technology and the increasing knowledge about this disease, more NIID cases have been reported in many countries (Takahashi-Fujigasaki et al., 2016); however, compared with other neurodegenerative diseases, its incidence is still extremely low according to current case reports. The characterization of its clinical manifestations is symptom heterogeneity, including cognitive dysfunction, Parkinsonism-like behavior, peripheral neuropathy, cerebellar ataxia, tremor, gait instability, myotonia, involuntary movement, muscle weakness, seizure, and headache (Sone et al., 2016; Takahashi-Fujigasaki et al., 2016; Tian et al., 2019; Wang et al., 2019a). Similar to amyloid plaques in Alzheimer’s disease (AD) and α-synuclein aggregates in Parkinson’s disease (PD), intranuclear eosinophil inclusion bodies are the main characteristic pathological changes in the central and peripheral nervous systems, as well as in various organs (Takahashi-Fujigasaki, 2003). However, unlike AD and PD, which are most sporadic in the population, most recently reported NIID cases are familial (Sone et al., 2016).

Repeated GGC expansion in the 5′UTR of the NOTCH2NLC gene was identified as the causative mutation for NIID in Japan and China in 2019 (Ishiura et al., 2019; Sone et al., 2019; Tian et al., 2019). These findings provide a potential and important criterion for the diagnosis of NIID, owing to the rapidly increased number of reported NIID cases. However, some studies also reported that some symptoms or signs, such as essential tremor and leukoencephalopathy, are associated with GGC repeat expansion in the NOTCH2NLC gene (Okubo et al., 2019; Sun et al., 2020). Moreover, in many studies, patients previously diagnosed with AD, PD, or frontotemporal dementia (FTD) were found to have this GGC repeat expansion in the NOTCH2NLC gene (Sone et al., 2019; Jiao et al., 2020; Sun et al., 2020). Due to the heterogeneity of the clinical phenotypes of NIID, clinical diagnosis of NIID is still difficult. In this review, we summarize the advances in the clinical features, pathology, genetics, and diagnosis of NIID, as well as NOTCH2NLC-related repeat expansion disorders.

## Clinical symptoms of neuronal intranuclear inclusion disease

**FIGURE 1:**
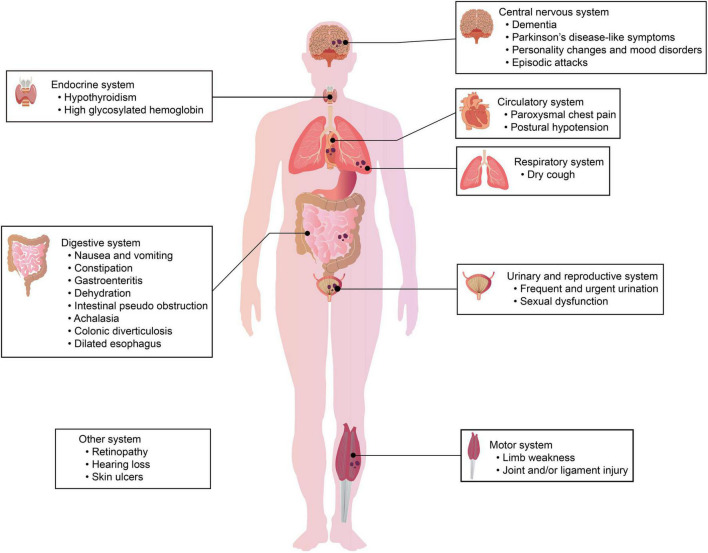
Clinical symptoms in different systems of patients with NIID. The clinical manifestations of NIID are highly heterogeneous. Patients with NIID may have symptoms/signs of the nervous system and/or symptoms/signs of other systems, including the respiratory system, urinary system, digestive system, and motor system.

Hundreds of NIID cases have been reported so far, and the most common feature of NIID is symptom heterogeneity; different families and individuals have different symptoms of NIID. In early clinical studies, the major clinical manifestations of previously described cases were cerebral cortical dysfunction and extrapyramidal symptoms, including cognitive dysfunction and dementia (Wang et al., 2020a; Zhang et al., 2020), PD-like behavior (Wang et al., 2020c), tremor (Kitagawa et al., 2014), ataxia (Imai et al., 2018), muscle weakness (Qin et al., 2021), bradykinesia, paroxysmal encephalopathy (Li et al., 2020b), and stroke-like episodes (Lin et al., 2020). However, except for nervous system symptoms, other symptoms, such as cough, vomiting (Okamura et al., 2020), retinal degeneration (Nakamura et al., 2020), and bladder dysfunction (Chen et al., 2020c), have been increasingly reported in many cases (Schuffler et al., 1978; Kimber et al., 1998; Horino et al., 2018). Here, we summarize the common nervous system symptoms and non-nervous system symptoms related to NIID, the details are presented in Figure 1 and Table 1.

**TABLE 1:** The clinical symptoms of NIID in different systems.

Systems	Symptoms and signs	References
**Nervous systems**	**Dementia-related symptoms**	
	Cognitive impairment	Kish et al., 1985; Goutieres et al., 1990; Lai et al., 2010; Aiba et al., 2016; Araki et al., 2016; Takeshita et al., 2017; Takumida et al., 2017; Hirose et al., 2018; Imai et al., 2018; Kawarabayashi et al., 2018; Nakamura et al., 2018; Liu et al., 2019b; Shindo et al., 2019; Yadav et al., 2019
	Dementia	Sung et al., 1980; Garen et al., 1986; Sone et al., 2014; Aiba et al., 2016; Araki et al., 2016; Abe and Fujita, 2017; Yamada et al., 2017; Nakamura et al., 2018; Chen et al., 2019; Yadav et al., 2019
	**PD-related symptoms**	
	Classic PD symptoms	Iizuka and Spalke, 1972; Kish et al., 1985; O’Sullivan et al., 2000; Lai et al., 2010; Wiltshire et al., 2010; Yoshimoto et al., 2017; Cupidi et al., 2019; Vermilion et al., 2019
	Tremor	Haltia et al., 1984; Kish et al., 1985; Goutieres et al., 1990; Afshari and Kamarei, 2005; Kitagawa et al., 2014; Yamaguchi et al., 2018; Yadav et al., 2019
	Ataxia	Lindenberg et al., 1968; Schuffler et al., 1978; Janota, 1979; Haltia et al., 1984; Soffer, 1985; Munoz-Garcia and Ludwin, 1986; Goutieres et al., 1990; Imai et al., 2018; Xiao et al., 2018; Yamaguchi et al., 2018
	Dystonia	Haltia et al., 1984; Paviour et al., 2005; Lai et al., 2010; Takumida et al., 2017; Chen et al., 2018; Kawarabayashi et al., 2018; Liu et al., 2019a; Vermilion et al., 2019
	Involuntary movements	Sung et al., 1980; Paviour et al., 2005; Takeshita et al., 2017; Hirose et al., 2018; Yamanaka et al., 2019
	Gait disturbance/dyskinesia	Janota, 1979; O’Sullivan et al., 2000; Wiltshire et al., 2010; Yamada et al., 2017; Yoshimoto et al., 2017; Horino et al., 2018; Xiao et al., 2018; Liu et al., 2019b; Yadav et al., 2019
	Hyporeflexia/hyperreflexia	Kimber et al., 1998; O’Sullivan et al., 2000; Afshari and Kamarei, 2005; Araki et al., 2016; Imai et al., 2018; Kawarabayashi et al., 2018; Nakamura et al., 2018; Liu et al., 2019a,b
	**Mood-related symptoms**	
	Emotional lability	Sung et al., 1980; Haltia et al., 1984; Lai et al., 2010; Wiltshire et al., 2010; Hirose et al., 2018; Kawarabayashi et al., 2018; Yamaguchi et al., 2018; Vermilion et al., 2019
	Abnormal behaviors	Goutieres et al., 1990; Sone et al., 2014; Han et al., 2019; Wang et al., 2019b
	**Others**	
	Muscle wasting	Malandrini et al., 1998; Yoshimoto et al., 2017; Omoto et al., 2018; Pilson et al., 2018; Xiao et al., 2018
	Paroxysmal encephalopathy	Xiao et al., 2018; Wang et al., 2019b
	Recurrent headaches	Han et al., 2019; Qin et al., 2020
	Sensory disturbance	Aiba et al., 2016; Araki et al., 2016; Sakurai et al., 2016; Abe and Fujita, 2017; Takeshita et al., 2017; Takumida et al., 2017; Nakamura et al., 2018; Omoto et al., 2018; Yamanaka et al., 2019
	Epileptic episodes	Haltia et al., 1984; Patel et al., 1985; Pilson et al., 2018; Shindo et al., 2019; Vermilion et al., 2019
**Circulatory system**	Cardiomyopathy	Oyer et al., 1991; Takumida et al., 2017; Yoshimoto et al., 2017; Yadav et al., 2019
	Coronary atherosclerosis	Parker et al., 1987
	Orthostatic hypotension	Aiba et al., 2016; Araki et al., 2016; Sakurai et al., 2016; Nakamura et al., 2018; Liu et al., 2019a; Vermilion et al., 2019
**Digestive system**	Nausea and vomiting	Barnett et al., 1992; El-Rifai et al., 2006; Xiao et al., 2018; Han et al., 2019; Shindo et al., 2019
	Constipation/diverticulosis	Lindenberg et al., 1968; Schuffler et al., 1978; Barnett et al., 1992; Aiba et al., 2016; Yamada et al., 2017; Vermilion et al., 2019
	Gastroenteritis	Lindenberg et al., 1968; El-Rifai et al., 2006
	Intestinal obstruction	El-Rifai et al., 2006; Yamaguchi et al., 2018
**Respiratory system**	Bronchopneumonia	Kimber et al., 1998; Wiltshire et al., 2010; Takumida et al., 2017; Pilson et al., 2018; Shindo et al., 2019; Vermilion et al., 2019; Qin et al., 2020
	Respiratory failure	Afshari and Kamarei, 2005; Paviour et al., 2005
	Respiratory distress	Malandrini et al., 1998
**Urinary and reproductive system**	Urinary dysfunction	Cupidi et al., 2019; Haltia et al., 1984; Han et al., 2019; Hirose et al., 2018; Horino et al., 2018; Imai et al., 2018; Kawarabayashi et al., 2018; Liu et al., 2019a; Nakamura et al., 2018; Shindo et al., 2019; Takumida et al., 2017; Yadav et al., 2019; Yamaguchi et al., 2018
	Sexual dysfunction	Zannolli et al., 2002; Han et al., 2019
	Glomerular lesion	Horino et al., 2018; Motoki et al., 2018
**Other systems**	Oculogyric crisis	Afshari and Kamarei, 2005; Wiltshire et al., 2010; Vermilion et al., 2019
	Pupillary dysfunction/miosis	Barnett et al., 1992; Kimber et al., 1998; Yamanaka et al., 2019
	Vision disorder/ptosis	Michaud and Gilbert, 1981; Chen et al., 2018; Hirose et al., 2018; Imai et al., 2018; Omoto et al., 2018; Hayashi et al., 2019; Yadav et al., 2019
	Nystagmus	Haltia et al., 1984; O’Sullivan et al., 2000; Lai et al., 2010; Imai et al., 2018; Liu et al., 2019a; Wang et al., 2019b
	Dysarthria	Janota, 1979; Tateishi et al., 1984; Patel et al., 1985; Malandrini et al., 1998; Takeshita et al., 2017; Imai et al., 2018; Nakamura et al., 2018
	Hearing losing	Soffer, 1985; Yoshimoto et al., 2017
	Dysphagia	Michaud and Gilbert, 1981; Tateishi et al., 1984; Malandrini et al., 1998; O’Sullivan et al., 2000; Afshari and Kamarei, 2005; Takumida et al., 2017; Chen et al., 2018; Vermilion et al., 2019

### Nervous system symptoms

#### Dementia

Dementia was one of the main symptoms described in a patient with NIID in 1978 (Schuffler et al., 1978). Since then, an increasing number of NIID patients with dementia have been reported, and patients with dementia are considered to be a subtype group of patients with NIID (Tian et al., 2019). Sone et al. (2016) analyzed the clinical features in 54 patients with NIID and found that dementia was the most prominent initial symptom in sporadic adult-onset NIID and familial NIID patients who are more than 40 years old. Araki et al. (2016) performed neuropsychological assessments in patients with NIID and revealed that language and executive functions were more prominent in these patients with dementia. In these patients, brain magnetic resonance imaging (MRI) showed leukoaraiosis and global cortical atrophy, especially in the cingulate and the temporal cortex regions (Cupidi et al., 2019). Moreover, a decline in the Mini-Mental State Examination score was found in both sporadic and familial NIID cases (Sone et al., 2016). Some patients previously diagnosed with AD were found to have GGC repeat expansions in the NOTCH2NLC gene (Tian et al., 2019), implicating that NIID should be considered as a differential diagnosis of AD.

#### Parkinson’s disease-like symptoms

Parkinson’s disease-like symptoms are regarded as another common and typical representative symptom in patients with NIID (Tian et al., 2019). PD-like symptoms in patients with NIID include resting tremors, rigidity, walking difficulty, clumsiness, and ataxia (Sone et al., 2016). Patients with PD-like symptoms usually respond well to levodopa treatment but easily develop dopa-induced motor fluctuations (O’Sullivan et al., 2000; Vermilion et al., 2019). Typical NIID pathology was found in some families with symptoms of PD (Tian et al., 2019). Recently, more groups found that patients with a diagnosis of PD or essential tremor have expended GGC repeats in the NOTCH2NLC gene (Tian et al., 2019; Ma et al., 2020; Sun et al., 2020; Shi et al., 2021). However, some groups showed that PD-like symptoms were the early-stage symptoms of NIID (Chen et al., 2020b; Yang et al., 2021). Therefore, PD-like symptoms are the dominant phenotypes of NIID (Huang et al., 2021). Patients with familial PD or essential tremors should be considered for the diagnosis of NIID or NOTCH2NLC gene-related disorders.

#### Personality changes and mood disorders

It is worth noting that a significant proportion of patients with NIID have adverse changes in terms of mood and personality (Sone et al., 2016; Chen et al., 2020a), but due to the non-specificity of these symptoms, personality changes and mood disorders are usually ignored in patients with NIID. Many patients with familial NIID gradually develop emotional instability, such as apathy, irritability, depression, and anxiety (Chen et al., 2020a). A previous case report also showed the presence of behavioral abnormalities in patients with NIID. Malandrini et al. (1996) reported a patient with NIID confirmed by a rectal biopsy having episodes of rage and aggressiveness that preceded other symptoms. Vermilion et al. (2019) reported that a NIID patient developed depression and anxiety at the age of 11 years, social isolation at 16 years, and increasing impulsivity at 17 years. Kawarabayashi et al. (2018) described a patient with NIID with the acute onset of apathy, and symptoms existed for a long time. In a recent study, approximately 60.8% of patients with NIID identified with genetic tests had different extents of abnormal behaviors, such as irritability, anxiety, depression, obsession, and impulsivity (Chen et al., 2020a). Based on the pathological and imaging results in patients with NIID (Sone et al., 2016; Chen et al., 2020a), it is reasonable that diffuse brain damage by neuronal intranuclear inclusion causes behavioral abnormalities. Therefore, abnormal behaviors or mood disorders are an ignored but important sub-phenotype of NIID.

#### Peripheral nervous symptoms

Sone et al. (2016) showed the involvement of peripheral nerve injury in patients with NIID and found a delay in conduction velocity or a decrease in amplitude in the electromyogram of motor and sensory nerves. Hirose et al. (2018) proposed that abnormalities in nerve conduction velocity and somatosensory-evoked potentials may be the diagnostic basis of NIID. In addition, the reflexes in some patients with NIID were hyporeflexia (Hirose et al., 2018). A recent study reported a case of NIID with mitochondrial encephalomyopathy, lactic acidosis, and stroke (MELAS)-like episodes in chronic polyneuropathy, and this patient developed slowly progressing muscle weakness and paraesthesia in all extremities (Ishihara et al., 2020).

Limb weakness was found in many patients with NIID and is one of the key phenotypes of NIID (Sone et al., 2016; Chen et al., 2020a). A previous study reported that a NIID patient had dyskinesia and upper limb chorea and could not walk or sit up without support (Pilson et al., 2018). Tian et al. (2019) demonstrated that the average onset age of muscle weakness in NIID patients with initial clinical manifestations was approximately 36 years. Muscle weakness tends to begin in the distal lower limbs and then move to the throat muscles and face. Recent studies have shown that many diseases with limb weakness, such as amyotrophic lateral sclerosis and oculopharyngodistal myopathy, are associated with NIID or NOTCH2NLC-related GGC repeat expansion disorders (Ogasawara et al., 2020; Yuan et al., 2020; Jih et al., 2021; Sugiyama et al., 2021). A recent clinical study indicated that 62.7% of patients with NIID had muscle weakness (Chen et al., 2020a). Due to the high occurrence of limb weakness in patients with NIID, the limb weakness-dominant subtype was proposed as one of the clinical phenotypes of patients with NIID (Sone et al., 2016).

#### Episodic attacks

Although progressive symptoms, such as cognitive impairment and parkinsonism, are key features of NIID as a neurodegenerative disease, episodic attacks are also found in some patients with NIID, such as encephalitis-like, vestibular migraine-like attacks, or MELAS-like or epileptic episodes (Xiao et al., 2018; Yamanaka et al., 2019; Ishihara et al., 2020; Li et al., 2020b; Ataka et al., 2021; Zhao et al., 2021).

Han et al. (2019) reported a 63-year-old male with NIID who had recurrent acute encephalopathy syndrome, including recurrent headaches, personality changes, and abnormal mental behavior for 3 years. Shindo et al. (2019) reported that a 65-year-old patient with NIID had recurrent paroxysmal nausea and vomiting that lasted 2–3 days for each episode, and this patient later developed a non-convulsive epileptic status with generalized periodic electrical discharge identified by an EEG test. In addition to case reports, Sone et al. (2016) found that sporadic patients with NIID had generalized convulsions (13.2%), disturbance of consciousness (39.5%), and encephalitic episodes (21%). These findings demonstrated that episodic attacks are common symptoms in patients with NIID. Recently, Wang et al. (2020b) proposed that episodic encephalopathy prior to other neurological symptoms was a valuable diagnostic indicator for adult-onset NIID.

#### Other symptoms in the nervous system

Several ophthalmological manifestations have been reported in patients with NIID, such as abnormal pupillary functions, miosis, oculogyric crisis (Vermilion et al., 2019), reduced eye movements, nystagmus, blepharospasm (Ogasawara et al., 2020), ptosis (Ogasawara et al., 2020), and loss of pigment in the retinal pigment epithelium (Haltia et al., 1986; Arrindell et al., 1991; Yamada et al., 2017). Pupillary dysfunction was reported to be a sensitive indicator of NIID (Arrindell et al., 1991). Adult-onset NIID patients with CGG repeat expansion in the NOTCH2NLC gene had similar ophthalmological characteristics, including rod-cone dysfunction with progressive retinal degeneration in the peripapillary and midperipheral regions; the most common symptoms in these patients were reduced visual acuity and night blindness (Nakamura et al., 2020). Therefore, retinal dystrophy was proposed as a NOTCH2NLC-related GGC repeat expansion disorder (Hayashi et al., 2020; Nakamura et al., 2020). In addition, pupil constriction (56.9%) and neurogenic bladder (23.5%) were found to be very common symptoms in patients with NIID and were considered to involve the autonomic nervous system (Chen et al., 2020a).

### Non-nervous system symptoms and signs

Except for the nervous system, many studies have indicated that other systems are involved in the pathology of NIID (Sone et al., 2016; Chen et al., 2020a). The non-nervous system manifestations of NIID make the diagnosis of NIID more difficult.

#### Respiratory system symptoms

A recent study showed that ubiquitin and p62-positive cells are found in the lung tissues of patients with NIID, indicating the presence of intranuclear inclusion bodies in the lung (Chen et al., 2020a). By interviewing the symptoms of patients with NIID, approximately 78.4% of patients had respiratory system symptoms, and the most common symptoms were intractable irritant dry cough (51.0%) (Chen et al., 2020a). Among these patients, 89.5% had positive chest CT results, and chronic inflammation signs, lung nodules, and interstitial changes were found in chest CT (Chen et al., 2020a). Lung biopsy showed the infiltration of neutrophil monocytes in the pulmonary interstitium, suggesting chronic inflammation in the lungs of patients with NIID (Chen et al., 2020a). In an overview of all the NIID-reported cases, a surprising and unexpected finding was that some reported patients eventually died of respiratory diseases, such as respiratory distress (Malandrini et al., 1998), bronchopneumonia (Kimber et al., 1998), or aspiration pneumonia (Pilson et al., 2018; Vermilion et al., 2019); however, the underlying mechanisms are still unclear.

#### Circulatory system symptoms

Cardiomyopathy and coronary atherosclerosis are reported to be associated with circulatory symptoms in patients with NIID. Oyer et al. (1991) found the presence of cardiomyopathy with intranuclear inclusions in myocytes in a NIID patient confirmed on postmortem, which expanded the known pathological spectrum of NIID. Parker et al. (1987) reported a 23-year-old patient with NIID with severe premature coronary atherosclerosis but no known risk factors. A recent study showed that approximately 72.5% of patients had circulatory system symptoms and signs (Chen et al., 2020a). In this study, paroxysmal chest pain (35.3%) and postural hypotension (29.4%) were common symptoms in the circulatory system (Chen et al., 2020a). Some non-specific changes in electrocardiograms, such as T wave or ST-T changes and atrial or ventricular premature beats, were found in patients with NIID. Cells in blood vessels from different tissues were found to be ubiquitin- and p62-positive (Chen et al., 2020a), suggesting the presence of intranuclear inclusions in the blood vessels.

#### Urinary system symptoms

In previous studies, intranuclear inclusions were found in the kidneys of patients with NIID (Horino et al., 2018; Motoki et al., 2018; Nakamura et al., 2018), but urinary system symptoms were not obvious. A case report showed an eosinophilic intranuclear inclusion in a renal biopsy obtained 12 years prior to the diagnosis of NIID, suggesting the possibility that the formation of intranuclear inclusions in kidneys may occur prior to neuronal degeneration for years (Motoki et al., 2018). Clinical symptoms and laboratory examinations by a clinical evaluation demonstrated the involvement of the urinary system in 66.7% of patients; common clinical manifestations, such as frequent and urgent urination, were found in 49.0% of patients with NIID (Chen et al., 2020a). Renal function insufficiency and abnormal urine routine tests were found in some patients with NIID; intranuclear inclusions detected by p62 and ubiquitin antibodies were observed in kidney and bladder tissues (Chen et al., 2020a). Bladder biopsy in three cases showed diffuse inflammatory cell infiltration, suggesting an inflammatory response by intranuclear inclusions in NIID (Chen et al., 2020a). A recent urodynamic report demonstrated bladder dysfunction in patients with NIID, including detrusor overactivity, decreased bladder sensation, and large post-void residual urine (Aiba et al., 2020). Current evidence shows the involvement of the urinary system in NIID.

#### Digestive system symptoms

Digestive system symptoms in patients with NIID commonly manifest as severe nausea and vomiting, which can occur alone, but in most cases, nausea and vomiting occur together. A case report showed that a patient with NIID repeatedly vomited for 7 years before the apparent abnormality in DWI was found, suggesting that periodic vomiting could be the only symptom of NIID in the early stages of NIID (Okamura et al., 2020). Gastrointestinal symptoms that presented in patients with NIID included bouts of constipation, gastroenteritis, dehydration, intestinal pseudo-obstruction, achalasia, colonic diverticulosis, and dilated esophagus (Barnett et al., 1992). Two siblings of patients with NIID were reported to present primary gastrointestinal dysfunction, such as abdominal pain, distention, and vomiting for 40 years (Schuffler et al., 1978). A systemic clinical evaluation showed that approximately 64.7% of patients had digestive system symptoms, including nausea, vomiting, and constipation; a portion of patients (15.6%) had abnormal liver functions, and 83.3% of patients had gastrointestinal polyps (Chen et al., 2020a). Diffused ubiquitin- and p62-positive cells were found in esophageal, stomach, gallbladder, and rectal tissues (Chen et al., 2020a), indicating the high involvement of digestive organs in NIID.

#### Other system symptoms

The patients with familial NIID were reported to have erectile dysfunction beginning in the first or second decade of life (Zannolli et al., 2002). Chen et al. (2020a) reported that approximately 43.1% of patients with NIID had sexual dysfunction. Many cases have reported prostatic hyperplasia in over 60-year-old male patients with NIID (Sone et al., 2014; Araki et al., 2016; Yamada et al., 2017; Chen et al., 2020a). Joint and spine MRI/CT showed joint degeneration and joint and/or ligament injury in all patients (Chen et al., 2020a). Approximately one-third of patients with NIID have endocrine abnormalities, such as high glycosylated hemoglobin and hypothyroidism (Chen et al., 2020a). In addition, blurred vision, hearing loss, and skin ulcers are common symptoms in patients with NIID (Chen et al., 2020a).

#### The sequences of involvement in different systems

Although different systems and organs are involved in NIID, the sequences of involvement in different systems may vary in individual patients according to previous case reports; for example, the existence of intranuclear inclusions and symptoms in systemic organs, such as the stomach and kidneys, precedes the onset of nervous system symptoms in NIID case reports (Morimoto et al., 2017; Motoki et al., 2018). Therefore, the age of onset in different systems may indirectly reflect the involvement of different systems. In a recent study, the median onset age was used to evaluate the sequence of involvement of different systems (Chen et al., 2020a). The median onset age in different systems was as follows; the locomotor system (3 years), reproductive system (28.5 years), digestive system (30 years), circulatory system (38.5 years), respiratory system (40 years), nervous system (50 years), and urinary system (55 years) (Chen et al., 2020a). Therefore, the nervous system was affected by NIID much later than most other systems.

## Brain imaging features of neuronal intranuclear inclusion disease

**FIGURE 2:**
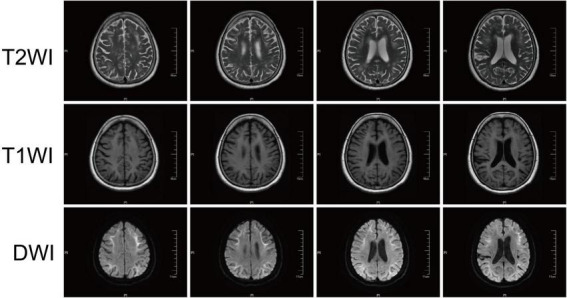
The typical brain magnetic resonance imaging (MRI) in patients with NIID. Curved or ribbon-like high signals along the corticomedullary junction, a strong clue for NIID, are found in the DWI sequence of head MRI of some patients with NIID.

Imaging examination is of great value in the clinical diagnosis of NIID. The earlier reported MRI findings were atrophy of the cerebellar hemispheres and no specific imaging for NIID (Zannolli et al., 2002). With the development of MRI technologies, high intensity of bilateral cerebral white matter on T2 and FLAIR, as well as a specific high-intensity signal in the corticomedullary junction on DWI (as shown in Figure 2) was found in patients with NIID identified by skin biopsy (Sone et al., 2014). These specific changes in the DWI sequence of MRI were further confirmed in a large number of patients with NIID and case reports (Sone et al., 2014, 2016; Chen et al., 2020a). Therefore, DWI high-intensity signals along the corticomedullary junction became a strong clue for the diagnosis of NIID (Sone et al., 2016). Usually, the DWI high-intensity signal in the corticomedullary junction extends with the disease worsening from a small regional portion in the frontal lobe to the cerebellum but does not expand into the deep white matter even with T2 widely expanded leukoencephalopathy (Sone et al., 2016); however, some cases also reported that the DWI high-intensity signal disappears several years later (Yokoi et al., 2016; Chen et al., 2018; Kawarabayashi et al., 2018). In the T2 flair sequence of MRI, except for abnormal intensity signals in the corticomedullary junction and periventricular areas, abnormal signals are also found in the callosum, cerebellum, and brainstem, indicating diffuse lesions in the brains of patients with NIID (Chen et al., 2020a). Recent studies also demonstrated that the high-intensity signal in the corticomedullary junction is consistent with the neuropathological findings that are spongiotic changes proximal to the U-fibers in subcortical white matter (Yokoi et al., 2016; Cupidi et al., 2019).

## Neuronal nuclear inclusions, the typical pathological changes in neuronal intranuclear inclusion disease

Neuronal nuclear inclusions (NIIs) in the skin or biopsy samples of other tissues are the characteristic histopathologic findings of NIID. NIIs are not limited to NIID, but are also present in a variety of multiple neurodegenerative diseases, such as fragile X-associated tremor/ataxia syndrome (FXTAS), distal ophthalmopharyngeal myopathy, and oropharyngeal myopathy, which have overlapping clinical symptoms and similar pathological outcomes, and even have some commonalities in genetic diagnosis (Boivin and Charlet-Berguerand, 2022; Ogasawara et al., 2022; Zhou et al., 2022). The existence of NIIs and the dysfunction of the ubiquitin-proteasome system (UPS) are the shared pathological features in NIID and other neurodegenerative diseases. Similar to these neurodegenerative diseases, the impairment of the UPS, such as the increase of ubiquitinated proteins and P62 protein, is found in the pathological changes of NIID (Oh et al., 2015; Hagerman and Hagerman, 2021; Boivin and Charlet-Berguerand, 2022; Ogasawara et al., 2022; Ribot et al., 2022; Zhou et al., 2022). However, how expanded GGC repeats cause the formation of NIIs and dysfunction of the UPS is generally unknown in the pathogenesis of NIID.

The NIID was defined as an intranuclear inclusion disease when it was first described in Lindenberg et al. (1968). In 1980, a case with progressive behavioral abnormalities, involuntary movements, ataxia, and dementia was reported, and pathological findings indicated that intranuclear inclusions were found in all types of central, peripheral, and autonomic neurons from almost all neuronal systems; therefore, this disease was proposed as NIID (Sung et al., 1980). Some studies confirmed the existence of NIIs in the cerebral cortex, basal ganglia, brain stem, and spinal cord, as well as in neurons and astrocyte glial cells (Greco et al., 2002; Zannolli et al., 2002; Takahashi-Fujigasaki, 2003; Nakamura et al., 2014; Sone et al., 2016; Yamaguchi et al., 2018). Intranuclear inclusions in glial cells were more commonly found in adult cases (Weidenheim and Dickson, 1995; Nakamura et al., 2014). However, later studies found that intranuclear inclusions involved multiple systems and not occurred only in the nervous system (Yamaguchi et al., 2018). Intranuclear inclusions have been observed in the following systems: the respiratory system, including the lungs (Yamaguchi et al., 2018); the gastrointestinal system, including the liver, spleen, pancreas, esophagus, stomach, jejunum, ileum, colon, and rectum (Sloane et al., 1994; El-Rifai et al., 2006; Mori et al., 2011); the endocrine system, including the parathyroid gland, pituitary gland, thyroid gland, and adrenal gland (Tateishi et al., 1984; Patel et al., 1985); the urinary system, including the kidney and urinary bladder (Motoki et al., 2018; Yamaguchi et al., 2018); the circulatory system, including the heart and lymph nodes (Oyer et al., 1991; Sone et al., 2016; Yamaguchi et al., 2018); the reproductive system, including ovaries and uterus (Yamaguchi et al., 2018); the locomotor system, including muscles (Morimoto et al., 2017); and the miscellaneous system, including the skin (Takumida et al., 2017; Hirose et al., 2018). A recent study systemically examined the distribution of NII detected by ubiquitin and P62 antibodies in tissue samples and found that NIID was in different systems except for the nervous system, indicating that NIID actually is a systemic intranuclear inclusion disease (Chen et al., 2020a). The spatial and temporal distribution in different systems may explain the highly heterogeneous phenotypes of NIID.

These inclusion bodies are circular, 1.5–10 μm in diameter, and are located near nucleoli, and ubiquitin/p62 staining, but not tau epitope, can be seen around NIIs (Bergmann et al., 1994; Takahashi et al., 2000). Electron microscopy showed a cluster of circular halo-shaped filamentous materials (8–12 nm) without limiting the membrane structure in the nucleus center (Deng et al., 2019). Intranuclear inclusions are considered to be formed when there is an excessive accumulation of proteins in the nucleus, and the abnormal alteration of nuclear bodies might be related to the pathogenesis of NIID (Takahashi et al., 2010; Nakano et al., 2017). Excessive protein accumulation in intranuclear inclusions might impair the ubiquitin-dependent degradation process and consequently result in the dysfunction of neurons or somatic cells (Liu et al., 2019a). The exact components of intranuclear inclusions are still unknown; previous studies have demonstrated that many nonspecific proteins, such as glucocorticoid receptor, promyelocytic leukemia protein (PML), histone deacetylase 4 (HDAC4), small ubiquitin modifier-1 (Sumo-1), fused in sarcoma, optineurin, myosin 6, heat shock protein 90, and dynamin-1, were found to be present in intranuclear inclusions by immunostaining (McFadden et al., 2005; Takahashi-Fujigasaki et al., 2006; Pountney et al., 2008; Nakamura et al., 2014; Nakano et al., 2017). The roles of these proteins and why these proteins are trapped in the intranuclear inclusions are still unclear and need further investigation.

Except for the NIIs in the different tissues, in the pathological examination of NIID tissues, diffuse inflammatory cell infiltration near the intranuclear inclusions, such as neutrophil monocytes and macrophages, was found in the affected tissues, including the brain, lung, bladder, and prostate gland (Chen et al., 2020a), indicating that inflammatory cell infiltration or inflammation in tissues are related to intranuclear inclusions. The inflammatory injury and edema can be seen in the brain from the DWI sequence of MRI as shown in the corticomedullary junction (Sone et al., 2014). In addition, edema was also found in the tissues of patients with NIID, supporting that dehydrate and anti-inflammatory drugs can be used for NIID treatment (Chen et al., 2020a; Liang et al., 2020). So far, a lack of clinical data supports the use of anti-inflammatory drugs in patients with NIID.

## Genetic and epigenetic progress of neuronal intranuclear inclusion disease

### NOTCH2NLC gene

In 2019, studies reported GGC repeat expansion at the 5′ region of NOTCH2NLC as the genetic cause of NIID (Deng et al., 2019; Sone et al., 2019; Tian et al., 2019). NOTCH2NLC is one of the three human-specific NOTCH2-derived genes (NOTCH2NLA, NOTCH2NLB, and NOTCH2NLC) on chromosome 1q21.1 and is highly expressed in the brain. The genomic sequences of the NOTCH2NL paralogs are similar to NOTCH2, including the NOTCH2 promoter and six N-terminal epidermal growth factor (EGF)-like domains from NOTCH2 exons 1 to 4 but without the transmembrane and cytoplasmic domains of NOTCH2 (Fiddes et al., 2018; Suzuki et al., 2018). These genes are considered to be involved in the evolutionary expansion of the human brain, and the mutations in these genes result in the reduction of brain size (Fiddes et al., 2018; Suzuki et al., 2018). NOTCH2NLC mRNA levels are unaltered in individuals with NIID (Ishiura et al., 2019; Sone et al., 2019; Tian et al., 2019), suggesting that GGC repeat RNA may change the functions of NOTCH2NLC mRNA/protein but not change the expression of NOTCH2NLC mRNA/protein to play an important role in the molecular pathogenesis of NIID.

Sun et al. (2020) identified that abnormal GGC repeat expansion in the 5′ region of the NOTCH2NLC gene is associated with essential tremor, which may explain tremor as a kind of symptom and sign of NIID in some relevant cases. Whether dynamic and/or postural tremors are the phenotypes of NIID requires a longer follow-up clinical and pathological examination (Chen et al., 2020b). Later studies demonstrated that many different diseases, such as amyotrophic lateral sclerosis, PD, dementia, oculopharyngodistal myopathy, leukoencephalopathy, and multiple system atrophy, were associated with GGC repeat expansion in the NOTCH2NLC gene (Okubo et al., 2019; Fang et al., 2020; Jiao et al., 2020; Ma et al., 2020; Ogasawara et al., 2020; Yuan et al., 2020). These studies showed two aspects: on the one hand, these diseases showed similar symptoms as NIID, indicating the symptom heterogenicity of NIID; on the other hand, similar to different variants of the same gene associated with distinct genetic diseases, different lengths of GGC repeat expansion in the NOTCH2NLC gene may cause different diseases with variable phenotypes, which are already reported in some studies (Sone et al., 2019; Tian et al., 2019). However, the association between GGC repeat size and different phenotypes is inconclusive (Huang et al., 2021). Therefore, to avoid confusion, NOTCH2NLC-related repeat expansion disorders were proposed to name symptom-heterogeneous diseases associated with GGC repeat expansion in the NOTCH2NLC gene (Westenberger and Klein, 2020).

Based on a multiethnic cohort of gene-confirmed patients with NIID from Southeast Asia, it was suggested that the presence of GGA interruptions in the repeated expansion of GGC may play a role in modifying the disease in terms of age at symptom onset (Chen et al., 2020c). Based on an in-depth study of NIID patients with European ancestry, GGC expansion in the NOTCH2NLC gene had a very low occurrence in Europe, suggesting that NOTCH2NLC repeat expansion is not the only cause of NIID onset or NII formation (Chen et al., 2020d); however, there was no evidence to support the hypothesis of gene heterogenicity of NIID (Li et al., 2020a).

### Epigenetic regulation of neuronal intranuclear inclusion disease

A previous study demonstrated that the NIIs in sporadic and familial NIID contained Sumo-1 and SUMOylation substrate PML and HDAC4 (Takahashi-Fujigasaki et al., 2006). Based on their results, both PML and Sumo-1 are major components of nucleosomes, suggesting that intranuclear inclusions in polyglutamine disease may originate from these functional domains that act as ubiquitin-related protein degradation sites. HDAC4 is also a major part of NIIs. HDAC is a transcriptional suppressor that regulates histone remodeling, and nucleosome is thought to be a site that controls histone acetylation levels. The presence of PML, Sumo-1, and HDAC4 in NIIs suggests that transcriptional activity regulated by histone acetylation may contribute to the disease process of NIID (Takahashi et al., 2010). In addition, previous studies also reported abnormal methylation in the CpG islands of the NOTCH2NLC gene (Tian et al., 2019; Deng et al., 2021). Hypermethylated CpG islands in the NOTCH2NLC gene were found in patients and asymptomatic carriers (Tian et al., 2019; Deng et al., 2021), suggesting that the hypermethylation status of the NOTCH2NLC gene may be related to the number of GGC repeats but not clinical symptoms. However, the mechanisms by which GGC expansion in the NOTCH2NLC gene influences methylation itself are still unclear (Tian et al., 2019).

## Possible mechanisms of neuronal intranuclear inclusion disease

**FIGURE 3:**
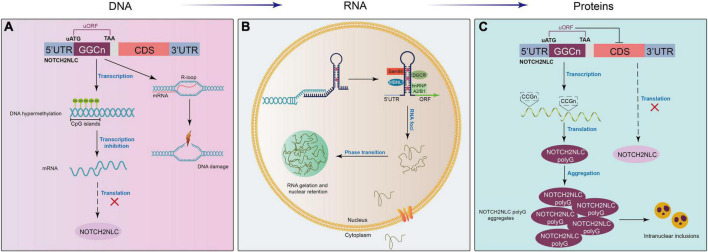
Proposed mechanistic models of NIID at different levels. **(A)** At the DNA level, expanded GGC repeats in the NOTCH2NLC gene induce the formation of RNA-DNA hybridization R-loops during transcription, promoting DNA damage and neuronal death. In addition, the hypermethylation of the NOTCH2NLC gene leads to gene silencing and the subsequent loss of protein function. **(B)** After transcription, extended GGC repeats sequester many RNA-binding proteins, forming RNA foci in the nucleus and resulting in the functional loss of RNA-binding proteins. **(C)** GGC repeat expansion initiates a near-homologous ACG codon located upstream of the GGC repeats in the NOTCH2NLC gene and is translated into the polyglycine (polyG)-NOTCH2NLC protein, which inhibits nucleocytoplasmic transport, leading to the accumulation of polyG-NOTCH2NLC and protein toxicity.

Although GGC repeat expansion in the NOTCH2NLC gene has been proven to be the cause of NIID, the molecular mechanisms underlying NIID remain unclear. Similar to FXTAS (GGC repeats in the FMR1 gene), Huntington’s disease (CAG repeats in the HTT gene), amyotrophic lateral sclerosis/FTD (GGGGCC repeats in the C9ORF72 gene), and myotonic dystrophy (DM1, CUG repeats in the DMPK gene), NIID is one kind of nucleotide repeat expansion disorder or microsatellite repeat expansion disorder with expanded GGC repeats in the NOTCH2NLC gene. Therefore, NIID may share some similar mechanisms to these microsatellite repeat expansion disorders. Here, we briefly propose some possible mechanistic models of NIID at the DNA, RNA, and protein levels (Figure 3). For more details, excellent review articles thoroughly discuss the various molecular mechanisms underlying pathogenesis in microsatellite diseases (Rodriguez and Todd, 2019; Boivin et al., 2021; Depienne and Mandel, 2021; Malik et al., 2021; Guo et al., 2022).

### Polyglycine protein toxicity

Unconventional translation, named repeat-associated non-AUG translation (RAN), of expanded repeats in toxic proteins has been identified in a variety of microsatellite disorders (Zu et al., 2011; Gao and Richter, 2017; Cleary et al., 2018; Cheng et al., 2019). For example, expanded GGC repeats located within the 5′UTR sequence of the FMR1 gene are embedded in a small upstream ORF (uORF), which is translated through initiation at a near-cognate ACG codon in a small polyglycine-containing protein, FMRpolyG (Krans et al., 2016, 2019; Sellier et al., 2017). The expression of FMRpolyG in cell and/or animal models forms protein inclusions and is toxic for neuronal cells, which could be associated with the presence of typical ubiquitin-positive intranuclear inclusions and neuronal cell death in the Fragile X Tremor Ataxia Syndrome (FXTAS) neurodegenerative disease (Glineburg et al., 2018; Hagerman and Hagerman, 2021; Zhao and Usdin, 2021). Similarly, two recent reports suggest that expanded GGC repeats embedded in the 5′UTR of the NOTCH2NLC gene are located in a small uORF, in which translation initiation starts at a canonical AUG codon, resulting in the expression of small polyglycine-containing proteins either named uN2CpolyG or N2NLCpolyG (Boivin et al., 2021; Zhong et al., 2021). Alike in FXTAS, the expression of uN2CpolyG/N2NLCpolyG forms protein aggregates and is toxic in cell and animal models. Moreover, antibodies directed against this polyglycine-containing protein stain the ubiquitin-positive intranuclear inclusions typical of NIID (Boivin et al., 2021; Zhong et al., 2021). Overall, these results may explain the origin of intranuclear inclusions and neuronal cell dysfunctions in NIID. Of interest, in both FXTAS and NIID, expanded CGG/GGC repeats are located in uORFs, in which translation starts ahead of the repeats and expression is independent of the downstream main FMRP or NOTCH2NLC proteins (Malik et al., 2021; Boivin and Charlet-Berguerand, 2022). Finally, these FMRpolyG and uN2CpolyG/N2NLCpolyG proteins were found to have liquid phase separation properties and impair nucleocytoplasmic transport, potentially illuminating putative molecular pathogenic mechanisms (Asamitsu et al., 2021; Zhong et al., 2021).

### RNA toxicity and RNA foci

Previous studies have demonstrated that repeat expansion-containing RNAs from the non-coding regions of genes linked to diverse human diseases, such as myotonic dystrophy type 1 and FXTAS, can form intramolecular hairpin secondary structures, bind to many RNA-binding proteins, and form RNA-protein aggregates in the pathology of these neurodegenerative diseases, resulting in toxic gain-of-function of these mRNAs (La Spada and Taylor, 2010; Xu et al., 2021). NOTCH2NLC mRNAs were supposed to have similar toxic gain-of-function; however, no evidence has shown the toxicity of NOTCH2NLC mRNAs in the pathology of NIID thus far. Recently, many reports have shown that these NOTCH2NLC mRNAs containing extended GGC repeats colocalize with p62 in nuclear inclusion bodies of patients with NIID, as well as in many RNA-binding proteins (Sam68, hnRNPA2/B1, MBNL1, DGCR, etc.) sequestered in the secondary structure of NOTCH2NLC mRNAs (Glineburg et al., 2018; Deng et al., 2021). These findings also indirectly support the RNA toxicity theory of NIID. Different repeat sizes, repeat locations, and sequestered RNA-binding proteins may influence RNA toxicity (Huang et al., 2021). Among them, sequestered RNA-binding proteins may play a critical role in RNA toxicity. Previous studies have shown that extended GGC repeats sequester many RNA-binding proteins and form RNA foci in the nucleus, resulting in the loss of RNA-binding protein functions and the occurrence of neurodegenerative diseases (Jain and Vale, 2017; Kong et al., 2017; Sanders and Brangwynne, 2017; Deng et al., 2021). RNA FISH technology combined with immunofluorescence demonstrated that RNA foci were observed in NIID-affected patients but not in the controls or asymptomatic carriers (Deng et al., 2021). However, the RNA-binding proteins that are specifically affected and their downstream signaling pathways need to be further identified.

### DNA damage and neuronal death

Extended GGC repeats may cause the pathology of neurodegenerative diseases not only at the protein level and the RNA level but also at the DNA level. A previous study indicated that GC enrichment in the extended GGC repeats of the FMR1 gene increases the propensity of the formation of RNA-DNA hybrid R-loops (Hamperl and Cimprich, 2014; Kong et al., 2017). These cotranscriptional R-loops can activate the DNA damage response and a series of signaling events, which result in DNA breakage and neuronal death (Barzilai, 2010; Kong et al., 2017). Due to similar extended GGC repeats in the NOTCH2NLC gene as in the FMR1 gene, extended GGC repeat-induced RNA-DNA hybrid R-loops and neuronal death may also contribute to the pathophysiology of NIID, which needs more investigation. In addition, recent studies have shown changes in the methylation level of the NOTCH2NLC gene (Deng et al., 2021; Huang et al., 2021). This methylation modification may also influence the transcriptional and translational levels that are linked to RNA toxicity and protein toxicity in the mechanisms of NIID. Therefore, different mechanisms of NIID may crosstalk or interact and contribute to the pathology of NIID.

## Diagnosis of neuronal intranuclear inclusion disease and differential diagnosis

### Diagnosis of neuronal intranuclear inclusion disease

**FIGURE 4:**
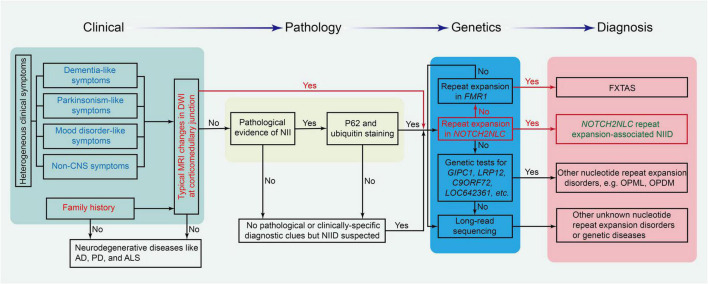
The flowchart for the diagnosis of NIID and its differential diseases. Different background colors in the chart show the four critical parts of the diagnosis protocol. AD, Alzheimer’s disease; ALS, amyotrophic lateral sclerosis; FXTAS, fragile X-associated tremor/ataxia syndrome; NII, neuronal intranuclear inclusion; PD, Parkinson’s disease.

In early case reports, autopsy, rectal biopsy, and nerve biopsy were mostly used to diagnose patients with NIID (Lindenberg et al., 1968; Funata et al., 1990; Goutieres et al., 1990; Zannolli et al., 2002; Yadav et al., 2019). Sone et al. (2011) found that the p62-positive nuclei of eosinophils detected by skin biopsy could be used for pathological diagnosis for NIID. Since then, many clinical cases have been confirmed by skin biopsy (Sone et al., 2014, 2016). With imaging development, the DWI sequence of head MRI showed a specific high signal along the corticomedullary junction (Sone et al., 2014). Therefore, the DWI imaging of head MRI became the key cue for the clinical diagnosis of NIID (Sone et al., 2016; Yu et al., 2019). After repeat GGC expansion in the NOTCH2NLC gene was confirmed as the cause of NIID in 2019 (Ishiura et al., 2019; Sone et al., 2019; Tian et al., 2019), genetic testing for GGC repeats in the NOTCH2NLC gene was performed and became the criteria for the diagnosis of NIID. The flowchart for the diagnosis of NIID and its differential diagnosis is shown in Figure 4.

Due to the high heterogeneity and multisystem symptoms of this disease, it is difficult to reach the diagnosis of NIID in clinical practice. The diagnosis of NIID can be considered from typical MRI signals, clinical manifestations, pathology of NIIs, and genetic tests. DWI high-intensity signals in head MRI are the strong indicators for the diagnosis of NIID. If patients had no typical MRI changes, the triad of clinical manifestations such as dementia, PD-like behaviors, and personality changes is a good cue for the diagnosis of NIID. For patients without the triad of NIID and DWI high-intensity signals, NIIs in the pathological findings from skin or other tissues are also a cue to suspect the diagnosis of NIID. Once the diagnosis of NIID is suspected, a genetic test for GGC repeats in the NOTCH2NLC gene should be performed to confirm or rule out the diagnosis of NIID, as shown in Figure 4.

### Differential diagnosis of neuronal intranuclear inclusion disease

Because NIID is a symptom-heterogeneous disease and shares similar imaging features with some diseases, NIID has many differential diagnoses from other diseases.

Because FXTAS and NIID are both autosomal-dominant GGC trinucleotide repeat expansion diseases in different genes (FMR1 gene and NOTCH2NLC gene, respectively) (Padilha et al., 2018; Toko et al., 2021), FXTAS is an important disease to differentiate from NIID. More importantly, FXTAS has similar clinical symptoms and radiological features to late-onset NIID, such as ataxia, tremor, Parkinsonism, cognitive decline, and bilateral high-signal abnormalities along the corticomedullary junction in DWI sequences (Leehey, 2009; Padilha et al., 2018). Therefore, genetic tests for the GGC repeats in the FMR1 gene and NOTCH2NLC gene may be a suitable method to distinguish FXTAS and NIID.

Neuronal intranuclear inclusion disease should be distinguished from Creutzfeldt–Jakob disease and some diseases with leukoencephalopathy or dementia. Creutzfeldt–Jakob disease has progressive cognitive impairment and myoclonus in the extremities, as well as characteristic lacy high-intensity signals in the cortex, caudate nucleus, or putamen on brain DWI. A recent study proposed that brain DWI is the key to differentiate from Creutzfeldt–Jakob disease and different leukoencephalopathy grades (Tokumaru et al., 2021).

Middle cerebellar peduncle lesions are considered to be a characteristic finding of NIID (Okamoto et al., 2003), but they can be found in other neurodegenerative disorders, such as multiple system atrophy and spinocerebellar ataxia, and diseases due to other etiologies (neoplasm, metabolic, cerebrovascular, inflammatory, and demyelinating diseases). Many neurodegenerative diseases, such as progressive supranuclear palsy, corticobasal degeneration, dementia with Lewy bodies, Perry syndrome, Huntington’s disease, dopa-responsive dystonia, Wilson disease, and neurodegeneration with brain iron accumulation, have PD-like behavior. Therefore, sometimes it is very difficult to differentiate these diseases from clinical symptoms. High-intensity signals along the corticomedullary junction on DWI at the late stage may also help clinicians distinguish NIID from the above neurodegenerative diseases and gelsolin amyloidosis, which can present with neuropathy, ataxia, and dementia (Pihlamaa et al., 2012).

## Treatment of neuronal intranuclear inclusion disease

Currently, there is no treatment to cure or slow down the process of NIID, but medications that control symptoms, such as muscle weakness, impaired consciousness, abnormal behavior, and sensory impairment, can improve a patient’s quality of life (Raza et al., 2020). Clinical treatment methods mainly adopt symptomatic treatment (Shindo et al., 2019). According to previous case reports, most patients with NIID are responsive to symptomatic treatment; for example, patients with NIID with Parkinsonism are sensitive to levodopa treatment (Lai et al., 2010; Ma et al., 2020), and patients with NIID with seizures are relieved by intravenous phenytoin and then carbamazepine (Fujita et al., 2017). However, some patients may experience relapse after effective treatment and/or develop other symptoms (Imai et al., 2018; Liu et al., 2019a; Li et al., 2020b). Because previous NIID cases eventually died of respiratory diseases, such as bronchopneumonia or aspiration pneumonia (Kimber et al., 1998; Malandrini et al., 1998; Pilson et al., 2018; Vermilion et al., 2019), it is necessary to prevent pulmonary infection and respiratory aspiration, particularly in patients with disturbed consciousness. Because diffuse inflammatory cell infiltration and edema were found in different tissues containing intranuclear inclusions (Chen et al., 2020a), dehydrate and anti-inflammatory drugs could be used to relieve the inflammation in patients with NIID, particularly in those with episodic attacks. In addition, nutritional support and psychotherapy should be provided during clinical treatments.

Recently, different therapeutic strategies have been proposed for NIID, for example, antisense oligonucleotide therapy, RNA interference, small molecule RNA drugs, and CRISPR-based therapy (Xu et al., 2021). Although these strategies are promising, the major challenge is the low specificity and high possibility of out-of-target therapies.

## Conclusion and future directions

With an increasing number of reported cases of NIID, the clinical manifestations of NIID are well characterized. Early studies showed that NIID mainly has neurological symptoms; the latest studies demonstrated that there are a variety of symptoms and signs in various systems, indicating the high heterogeneity of clinical symptoms and signs of NIID. This increases the difficulty in the clinical diagnosis of NIID. Although patients with NIID present heterogeneous symptoms, they also show some core symptoms, such as triad symptoms in the central nervous system (dementia, Parkinsonism, and psychiatric symptoms). Before the NOTCH2NLC gene was linked to the etiology of NIID, tissues/skin biopsy and DWI high-intensity signals along the corticomedullary junction were strong clues for the diagnosis of NIID; now, genetic tests for expanded GGC repeats in the NOTCH2NLC gene have become the gold standard for NIID.

Expanded GGC repeats in the NOTCH2NLC gene have been confirmed as the cause of NIID, but how expanded GGC repeats in the NOTCH2NLC gene cause pathological changes and the accumulation of NII in the nucleus remains unclear. As a nucleotide repeat expansion disorder, NIID may share similar mechanisms to other nucleotide repeat expansion disorders, such as expanded nucleotide repeat-induced DNA damage, RNA toxicity, and abnormal encoded protein toxicity. Although recent studies have demonstrated the presence of polyG-NOTCH2NLC protein in the animal models and tissues of patients with NIID, the mechanism by which the polyG-NOTCH2NLC protein causes toxicity still needs more investigation. RNA toxicity and DNA instability theory need to be identified in the mechanisms of NIID. How do expanded GGC repeats in the NOTCH2NLC gene induce the formation of NIIs? What are the components in expanded GGC repeat-induced RNA foci? Which RNA-binding proteins are involved in the pathogenesis of NIID? These questions await more investigation, and the answers to these questions will help to guide the treatment of NIID. To date, no effective treatment is available. Even though different gene therapies, such as antisense oligonucleotide therapy and RNA interference, have been proposed, many great challenges must be overcome, such as the specificity and out-of-target nature of RNA drugs. Recent findings have shown that inflammation is involved in the pathological changes of NIID and may be downstream of expanded GGC repeat-induced RNA/protein toxicity. Therefore, the role of inflammation should be further identified in the pathology of NIID, and anti-inflammation could be a promising therapeutic strategy for NIID.

## Author contributions

XX, MS, and BoW designed and revised the manuscript. YL and HL collected the literature and made the first draft. XL, BiW, HY, BoW, and MS discussed and interpreted the analysis. All authors contributed to the article and approved the submitted version.
